# Cellular and molecular mechanisms of alpha lipoic acid’s protective effects against diclofenac-induced hepatorenal toxicity

**DOI:** 10.2478/jvetres-2025-0029

**Published:** 2025-05-23

**Authors:** Hanan A. Ogaly, Neven Hassan, Reham M. Abd Elsalam, Shymaa El Badawy, Muhammad A. Alsherbiny, Bardes Hassan, Fatimah A.M. Al-Zahrania, Gehan Othman, Chun Guang Li, Sherif H. Elmosalamy

**Affiliations:** 1 Chemistry Department, College of Science, King Khalid University, Abha 61413, Saudi Arabia; 2 Department of Physiology, Faculty of Veterinary Medicine, Cairo University, Giza 12211, Egypt; 3 Department of Pathology, Faculty of Veterinary Medicine, Cairo University, Giza 12211, Egypt; 4 Department of Pharmacology, Faculty of Veterinary Medicine, Cairo University, Giza 12211, Egypt; 5 Pharmacognosy Department, Faculty of Pharmacy, Cairo University, Cairo 11562, Egypt; 6 Biology Department, College of Science, King Khalid University, Abha 61421, Saudi Arabia; 7 National Institute of Complimentary Medicine Health Research Institute, Western Sydney University, Westmead NSW 2145, Australia

**Keywords:** alpha lipoic acid, antioxidant, *Nrf2*, *NQO1*, *HO-1* genes

## Abstract

**Introduction:**

The cellular and molecular pathways of α-lipoic acid’s (ALA’s) protective effect were assessed against diclofenac (DIC) hepatorenal injury *in vivo* and against a pro-inflammatory stimulus *in vitro*.

**Material and Methods:**

The injury was induced in 28 adult male Wistar rats weighing 130–160 g by a single intraperitoneal injection of DIC (50 mg per kg body weight (b.w.)) on the fifth day. Seven positive control rats had received no hepatorenally protective compounds. Oral 100 mg/kg b.w. doses of silymarin (SLY) were given to seven animals, 50 mg/kg b.w. doses of ALA to seven more and 100 mg/kg b.w. doses of it to another seven for five days before DIC insult. Seven negative control rats received only distilled water instead of protective compound and in the injection. The anti-inflammatory effect of ALA was also assayed in murine RAW264.7 macrophage cells.

**Results:**

In the cells, ALA was antioxidant and anti-inflammatory in a dose-dependent manner, reducing nitric oxide (NO) and reactive oxygen species generation with half maximal concentrations of 7.8 and 6.25 μg/mL, respectively. Both ALA doses and SLY protected the hepatorenal tissues and improved kidney and hepatic functions compared to the organs of the positive control group. Additionally, ALA reduced oxidative stress biomarker levels in hepatic and renal tissues compared to the positive control rats. It also improved liver and kidney histology, where hepatic lesions were fewer, and protected renal architecture. Immunohistochemical analysis showed ALA to reduce caspase-3 expression, supporting its hepatorenal anti-apoptotic effect. Alpha lipoic acid markedly upregulated the hepatorenal messenger RNA expressions of nuclear factor erythroid 2-related factor 2 (*Nrf2*), haem oxygenase-1 and nicotinamide adenine (phosphate) reduced form : quinone oxidoreductase 1, suggesting that the Nrf2 signalling pathway was enhanced.

**Conclusion:**

These findings suggested potential therapeutic benefits for ALA in mitigating DIC-induced hepatorenal toxicity through its anti-inflammatory, antioxidant and Nrf2-mediating effects. Future investigations are warranted to explore the synergistic interactions and multiomics mechanisms.

## Introduction

Diclofenac (DIC) is primarily used as an antipyretic, anti-inflammatory and antinociceptive drug; besides this use, it is also commonly prescribed as a non-steroidal anti-inflammatory drug (NSAID) (32). Despite its therapeutic potential, DIC induces hepatorenal toxic side effects more frequently than other NSAIDs (2, 32). Upon cytochrome P450-dependent oxidation, metabolites of DIC initiate a rapid glutathione reduction, producing excessive reactive oxygen species (ROS) and causing mitochondrial redox imbalance (19). Also, ROS constantly cause lipid peroxidative damage in tissues *via* unsaturated fatty acid insult (14). Extra ROS-induced hepatocytic damage induces the secretion of inflammatory and proinflammatory cytokines, including tumour necrosis factor α, interleukin (IL)-1β and IL-6 (20). The interaction of metabolic and immunological factors determines individual susceptibility to DIC-induced idiosyncratic hepatotoxicity, where the cytokines that modulate the immune response and possess anti-inflammatory properties can mitigate hepatorenal damage induced by DIC (5). Consequently, it seems reasonable to assume that employing novel therapies can protect against DIC-induced hepatorenal damage *via* inhibition of the oxidative stress initiation and propagation, along with pro-inflammatory cytokine or pro-apoptotic gene downregulation (18).

Natural compounds may offer better remedies for NSAID-induced toxicity than current drugs that pose a danger of adverse effects (5). One example, the organosulphur compound α-lipoic acid (ALA), is a 1,2-dithiolane-3-pentanoic acid, is a naturally occurring essential co-enzyme that has recently drawn a lot of interest as a potent antioxidant and glutathione precursor, and is crucial for mitochondrial dehydrogenase processes (12). Typical sources of ALA in the diet are meat and, in lesser quantities, fruits and vegetables. Alpha-lipoic acid and its reduced form have been demonstrated to have scavenging activity against several intracellular free radicals, such as ROS and peroxide, hydroxyl and superoxide radicals (26). Cellular defence against oxidative stress relies on two major systems: enzymatic antioxidants, such as catalase (CAT), glutathione peroxidase and superoxide dismutase (SOD); and non-enzymatic antioxidants, including the reduced form of glutathione (GSH) (27). Alpha lipoic acid strengthens this defence in multiple ways: by being a precursor for GSH synthesis, acting as a cofactor for several mitochondrial enzymes including pyruvate dehydrogenase, and recycling other cellular antioxidants such as vitamin E (alpha tocopherol) and vitamin C (ascorbic acid) (12, 26). Administration of ALA is proven to be beneficial in several rodent oxidative stress models, such as neuroinflammation (15), polyunsaturated fatty acid hepatic injury (24), chemotherapeutic injury (10) and polycystic ovarian syndrome (12). However, the exact molecular mechanisms of DIC-induced hepatorenal toxicity and the beneficial effects of ALA have not yet been adequately understood. The primary aim of the current study is to assess ALA’s protective efficacy against hepatorenal damage induced by DIC in rats as well as its antioxidant and anti-inflammatory properties.

## Material and Methods

### Chemicals, therapeutic agents, diagnostic kits and cell culture materials

Chemicals of an analytical grade were used in the current study. Diclofenac sodium, silymarin (SLY) and ALA (CAS No. 1077-28-7) were obtained from Novartis Pharma (Basel, Switzerland), SEDICO Pharmaceuticals (6th October City, Egypt) and Sigma-Aldrich (St. Louis, MO, USA), respectively. Kits to measure the biochemical activity and oxidative stress parameters specific to aspartate aminotransferase (AST – cat. No. 260002), alkaline phosphatase (ALP – cat. No. AP 10 21), alanine aminotransferase (ALT – cat. No. 292002), total protein (cat. No. 310001), albumin (cat. No. 211001), uric acid (cat. No. UA 21 20), urea (cat. No. UR 21 10), total bilirubin (cat. No. BR 1110), creatinine (cat. No. CR 12 50), GSH (cat. No. GR 25 11), catalase enzyme (CAT – cat. No. CA 25 17) and malondialdehyde (MDA – cat. No. MD 25 29) were purchased from Bio Diagnostic (Dokki, Giza, Egypt). For anti-inflammatory activity assessment, Dulbecco’s modified Eagle’s medium (DMEM – Lonza, Basel, Switzerland), foetal bovine serum (FBS – Interpath, Somerton, VIC, Australia), penicillin and streptomycin (Sigma-Aldrich), glucose, L-glutamine and sodium pyruvate (Lonza), and murine RAW264.7 macrophage cells (American Type Culture Collection, Manassas, VA, USA) were obtained. For immunohistochemistry, rabbit polyclonal anti-caspase-3 (cat. No. A11319; ABclonal Biotech, Wuhan, China), horseradish peroxidase (HRP)-conjugated polyclonal goat anti-rabbit immunoglobulin G heavy and light chain (H&L) (cat. No. ab205718; Abcam, Cambridge, UK), streptavidin peroxidase (Thermo Scientific, Rockford, IL, USA) and 3,3'-diaminobenzidine tetra-hydrochloride (Sigma-Aldrich) were purchased.

### Experimental animals and laboratory diet

Adult male Wistar rats aged 5 to 6 weeks and weighing between 130 and 160 g were sourced from the Animal House Colony at VacSera (Giza, Egypt). They were housed in the laboratory facilities at the Faculty of Veterinary Medicine, Cairo University, Giza, Egypt. Prior to the experiment, the rats were acclimatised for one week and were kept under standard laboratory conditions, including a controlled room temperature of 22 ± 1°C, relative humidity of 54–68% and a 12-h light/dark cycle. They had unrestricted access to a balanced diet consisting of 1% vitamin mixture, 4% mineral mixture, 10% corn oil, 20% sucrose, 0.2% cellulose, 10.5% casein and 54.3% starch, along with fresh water.

### Experimental design

Thirty-five rats were randomly divided into 5 groups of seven rats each. Group I (negative control) rats received a daily oral dose of (1 mL) sterile distilled water for five days, followed by one intraperitoneal (I/P) sterile distilled water injection. Group II (DIC, positive control) rats received a daily oral dose of 1 mL distilled water for five days and were then subjected to a hepatorenal toxic insult through I/P injection of DIC in sterile distilled water at a dose of 50 (mg/kg body weight (b.w.)) 1 h after the last oral dose (31). Group III (SLY) rats were pretreated orally with SLY at a dose of 100 mg/kg b.w. daily as a standard hepatoprotective reference drug for five days and were then subjected to the same insult as previously described, also 1 h after their SLY dose (21). Group IV (ALA 50) rats were pretreated orally with ALA at a dose of 50 mg/kg b.w. daily for five days before hepatotoxicity induction as in the other groups (25). Group V (ALA 100) differed only from the ALA 50 group in the dose which they received, this being 100 mg/kg b.w. (12).

### Sample collection

At the end of the experiment, the animals were anaesthetised with I/P ketamine at 91 mg/kg b.w. 24 h after the last treatment. Collection of blood samples from the retro-orbital sinuses and their transfer to gel separator tubes to collect serum samples followed. The serum was collected by centrifugation of the blood at 3,000 × *g* at 4°C for 10 min and stored at –80°C to further determine biochemical and antioxidant biomarkers. The animals were weighed and euthanised by cervical dislocation. The liver and kidney tissues were removed, excess fat was trimmed, and then they were weighed. The tissues were divided into three portions, with two sets being stored at –80°C for quantitative reverse-transcription PCR (qRT-PCR) and antioxidant analysis. The final set of hepatorenal tissues was preserved in 10% neutral-buffered formalin for histological and immunohistochemical examination.

### Anti-inflammatory activity

Murine RAW264.7 macrophage cells were cultured in DMEM, which included 4.5 g/L of glucose, L-glutamine and sodium pyruvate, and had 5% FBS and 100 U/mL of penicillin and streptomycin added to it. The cells were maintained at 37°C in a 5% CO_2_ atmosphere until they achieved 90% confluency. At a seeding density of 0.85 × 106 mL^−1^, the cells were divided and plated into 96-well cell culture plates. They were then incubated for 24 h, rinsed with serum-free media and exposed to several treatments that had been researched, guided by prior research that demonstrated broad-spectrum anti-inflammatory and antioxidant effects of natural products, supporting the rationale for investigating similar activities of alpha-lipoic acid and diclofenac in this study. (9).

### *In vitro* anti-inflammatory assay (nitric oxide assay)

Lipopolysaccharides (LPS) at a concentration of 50 ng/mL stimulated RAW264.7 macrophage cells to generate nitric oxide. The NO was quantified as total nitrite content using freshly made Griess reagent as described previously (9, 22). In summary, after administering ALA at concentrations of 50, 25, 12.5, 6.25, 3.125, and 1.5625 μg/mL for 2 h, an inflammatory response was induced by the addition of lipopolysaccharide (LPS) at 50 ng/mL. The cells were then incubated for a further 24 h to evaluate the anti-inflammatory effects of ALA pre-treatment. A microplate spectrophotometer (CLARIOstar; BMG Labtech, Ortenberg, Germany) was employed to measure absorbance at 540 nm after 100 μL of the supernatant was collected and mixed with Griess reagents. The viability of the treated RAW264.7 macrophage cells was assessed using a 3-(4,5-dimethylthiazol-2-yl)-2,5-diphenyltetrazolium bromide (MTT) assay. After treatment and stimulation, the cells were co-incubated with MTT (0.12 mg/mL) for 4 h. Following the removal of the supernatant, the optical density was measured at 510 nm using the same microplate spectrophotometer. Cells which had not been stimulated by LPS (positive control cells) were used as a baseline for calculating the percentage of viable cells and nitric oxide (NO) compared with the negative control (untreated and unstimulated cells).

### Reactive oxygen species assay

The ROS assay was carried out on the same murine RAW264.7 macrophage cells used in the previous stages. It utilised a 2′,7′-dichlorofluorescein diacetate cellular ROS detection assay kit (cat No. ab113851; Abcam, Cambridge, UK), in accordance with the manufacturer’s guidelines and as described in our earlier studies. A positive control of 250 μM tert-butyl hydroperoxide was used. The fold increase in ROS generation was determined relative to the untreated control (cells treated with the buffer as specified in the procedure) (7).

### Biochemical analysis

Liver function was assessed *via* measurement of ALT, AST, ALP, total proteins, albumin and total bilirubin. Kidney function was also evaluated through the measurement of urea, creatinine and uric acid. All measurements were made with standardised kits purchased from Bio Diagnostic and used as directed in the manufacturer’s protocol.

### Oxidative stress biomarkers

Hepatic and renal tissues were mixed uniformly in cold buffered saline (10 mL/g tissue) with a glass homogeniser before being centrifuged at 4,000 rpm for 15 min at 4°C. The buffer for measuring MDA contained 50 mM potassium phosphate at pH 7.5, as did the buffer for GSH along with 1 mM ethylenediaminetetraacetic acid (EDTA). For CAT analysis, the buffer was composed of 50 mM potassium phosphate at pH 7.4, 1 mM EDTA and 1 mL/L Triton X-100 per gram of tissue. The concentrations of MDA, GSH and CAT were then assessed in the supernatants according to standard methods (3, 14).

### Histopathological studies

Livers and kidneys from different experimental groups were collected, preserved in 10% neutral-buffered formalin for 48 h, embedded in paraffin blocks, sliced into sections and stained with haematoxylin and eosin. Two veterinary pathologists rated sections blindly semi-quantitatively for hepatic (neuroinflammatory) and renal (glomerular, interstitial and vascular) abnormalities. The grading of the hepatic and renal lesions was performed applying previously described techniques (22).

### Immunohistochemistry

The tissue sections were dewaxed and rehydrated before their treatment with 10 mM citrate buffer for antigen retrieval. In a humidified chamber, the primary antibody, rabbit polyclonal antibody against caspase-3, was incubated in sections overnight at 4°C. The tissue sections were then treated for 10 min with goat anti-rabbit IgG H&L (HRP). Slides were treated with streptavidin peroxidase and 3,3'-diaminobenzidine tetra-hydrochloride and were then incubated for 10 min. After counterstaining with Mayer’s haematoxylin, the slides were dehydrated and mounted.

The primary antibodies were omitted in negative controls (27). A Qwin 500 Image Analyzer (Leica Microsystems, Wetzlar, Germany) was used to examine tissue sections.

The immunolabelled regions were determined in each field of stained sections. The positively stained area percentage (%) was measured by counting positive cells and the total number of cells was counted under a high-power field (×400) microscope in 10 microscopic fields/slides that were randomly selected.

### Relative gene expression analyses

Messenger RNA (mRNA) expression levels of Kelch-like erythroid cell-derived protein with cap 'n' collar homology– associated protein 1 (Keap1)/nuclear erythroid 2-related factor 2 (Nrf2) signalling pathway–related genes were determined in the hepatic and renal tissues using a qRT-PCR. Total cellular RNA from fresh liver and kidney tissue samples (approximately 100 mg) was isolated in ice-cold TRIZOL reagent according to the manufacturer’s technique (Invitrogen, San Diego, CA, USA). The yield and purity of the RNA were determined using a NanoDrop System (Thermo Fisher Scientific, Wilmington, DE, USA) at 260/280 nm wavelengths. A RevertAid cDNA Synthesis Kit (Thermo Fisher Scientific, Vilnius, Lithuania) was used with 1 μg RNA samples to reverse-transcribe them into cDNA. The relative mRNA expression levels of *Nrf2*, haem oxygenase 1 (*HO-1*) and nicotinamide adenine (phosphate) reduced form : quinone oxidoreductase 1 (*NQO1*) were determined using an ABI 7900HT Real-Time PCR System (Applied Biosystems, Foster City, CA, USA). The *β-actin* gene was the housekeeping gene. The oligonucleotide primers employed for qRT-PCR analyses are recorded in Table 1. Relative gene expression was calculated as fold-change difference after normalisation against the mRNA level of β-actin using the threshold-cycle formula (2^–△△CT^).

**Table 1.** Primers used for quantitative reverse-transcription PCR to determine nuclear factor erythroid 2-related factor 2 (Nrf2) pathway genes in liver and kidney tissue from Wistar rats subjected to hepatorenal toxic insult by diclofenac sodium (DIC) after being administered or denied protective compounds

Gene	Sense primer (5′–3′)	Antisense primer (5′–3′)	GenBank accession No.
*Nrf2*	CACATCCAGACAGACACCAGT	CTACAAATGGGAATGTCTCTGC	XM_006234398.3
*HO-1*	ACAGGGTGACAGAAGAGGCTAA	TCAAGAGGAGCAGAAAAAGAACAAG	NM_012580.2
*NQO1*	CTGTGAGGGACTCTGGTCTTTG	CTGAAAGCAAGCCAGGCAAAC	NM_017000.3
*β-actin*	GGTGGGTATGGGTCAG	ATGCCGTGTTCAATGG	NM_031144.3

1 *HO-1* – haem oxygenase-1; *NQO1* – nicotinamide adenine (phosphate) reduced form : quinone oxidoreductase 1

### Statistical analysis

The various analytical measurements were performed in triplicate, and the results were reported as the mean ± standard deviation (SD), where n = 7. GraphPad Prism software version 9.0 (GraphPad Software, San Diego, CA, USA) was used to determine the half maximal inhibitory concentration (IC_50_) and half maximal effective concentration of the tested drugs, which involved performing a nonlinear regression of the log-transformed concentration’s variable slope (four parameters) against the normalised response. Mean difference values in the experiments at P-value < 0.05 were considered statistically significant. One-way or two-way analysis of variance was performed to compare the means using *post hoc* Tukey’s corrections for multiple comparisons or a Kruskal–Wallis test followed by a Dunn’s test for the histopathological scoring, or a Tukey multiple-comparisons test for all other experiments at 95% confidence.

## Results

### *In vitro* anti-inflammatory and antioxidant activity

**Fig. 1.**
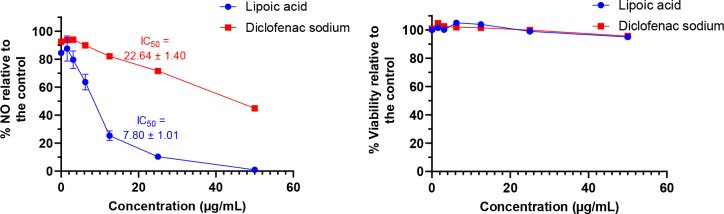
The inhibitory effect on murine RAW264.7 macrophage cells of different α-lipoic acid and diclofenac sodium concentrations on A) nitric oxide (NO) production and B) cell viability assayed by 3-(4,5-dimethylthiazol-2-yl)-2,5-diphenyltetrazolium bromide (n = 3). Data was expressed as mean ± standard deviation. IC_50_ – half maximal inhibitory concentration; ** and **** – significant difference relative to the negative control at P-value < 0.01 and P-value < 0.0001, respectively

The anti-inflammatory activity of ALA at 50ug/mL and lower concentrations in six different 1 : 2 serially dilutions against the effect of DIC relative to the control was assessed using a nitric oxide assay (Fig. 1A). Inhibition of NO production by 50% was observed at 7.8 ± 1.01 and 22.64 ± 1.4 μg/mL (mean ± SD) for ALA and DIC, respectively. A dose-dependent decline in NO production was exhibited for ALA and DIC with a significant difference (P-value < 0.05) relative to the control down to 6.25 and 25 μg/mL, respectively (Fig. 1A). The different concentrations showed cell viability above 90% relative to the negative control as measured by the MTT assay, indicating that the NO-depletive activity is related to the drug treatment rather than the cytotoxicity (Fig. 1B).

### Positive ROS control

**Fig. 2.**
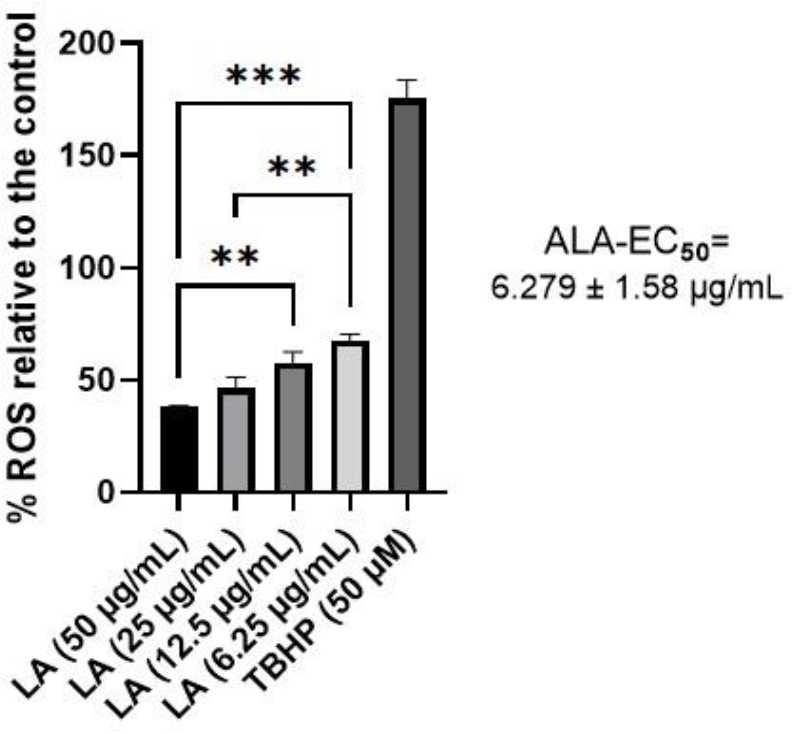
The inhibitory effect in murine RAW264.7 macrophage cells of different alpha lipoic acid (ALA) and diclofenac sodium concentrations on reactive oxygen species (ROS) production. Data are expressed as mean ± standard deviation, n = 3. EC_50_ – half maximal effective concentration; TBHP – tert-butyl hydroperoxide; ** and *** – significant differences at P-value ≤ 0.005 and 0.0005, respectively

Tert-butyl hydroperoxide at 50 μM increased the ROS production to 175.25 ± 8.28% (Fig. 2). Alpha lipoic acid produced a significant dosedependent inhibition of ROS production at 50, 25, 12.5 and 6.25 μg/mL concentrations relative to the control, with IC_50_ of 6.279 ± 1.58 μg/mL (Fig. 2).

### Biochemical analysis

Liver function markers (ALT, AST, ALP and total bilirubin) and kidney function markers (urea, creatinine and uric acid) were significantly elevated in the DIC-challenged and hepatorenally unprotected group relative to the negative control group. Serum total proteins, albumin and globulin concentrations showed a significant reduction in the DIC-challenged and hepatorenally unprotected group compared with the negative control group. The pre-administration of SLY, ALA at 50 mg/kg b.w. and ALA at 100 mg/kg b.w. revealed a substantial protective effect, and less damage from the insult than in the unprotected DIC group. This protection was revealed through a substantial inhibition of liver enzymes (AST, ALT and ALP), total bilirubin and kidney function markers (urea, creatinine, and uric acid), and elevation of total protein, albumin and globulin concentrations in comparison with the DIC-challenged group. The group receiving the higher dose, ALA 100, showed the best improvement when compared with the other pretreated groups (Table 2).

**Fig. 3.**
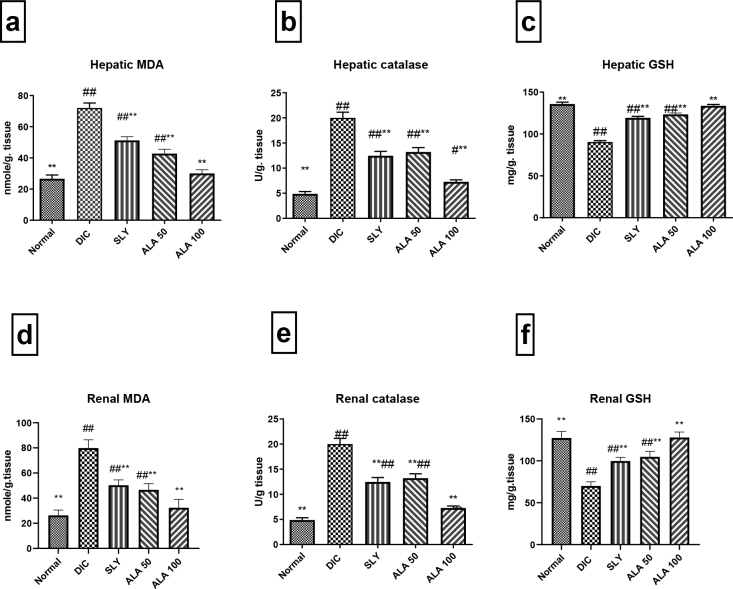
The effect of hepatoprotective compounds on hepatic and renal oxidative biomarkers in Wistar rats subjected to hepatorenal toxic insult by diclofenac sodium (DIC) and contrast in rats denied the compounds. A and D – effect on malondialdehyde (MDA); B and E – effect on catalase (CAT); C and F – effect on reduced glutathione (GSH). Data are expressed as mean ± standard deviation. n = 7 rats/group. * and ** – significant difference *vs* negative control group at P-value ≤ 0.05 and P-value ≤ 0.005, respectively; ^#^ and ^##^ – significant difference *vs* DIC group at P-value ≤ 0.05 and P-value ≤ 0.005, respectively; SLY – silymarin at 100 mg/kg body weight (b.w.); ALA 50 – α-lipoic acid at 50 mg/kg b.w.; ALA 100 – α-lipoic acid at 100 mg/kg b.w.

**Table 2.** Biochemical analysis of liver and kidney function markers in in Wistar rats subjected to hepatorenal toxic insult by diclofenac sodium (DIC) after being administered or denied protective compounds

Biomarker	Group					Biomarker	Group				
	Control	DIC	SLY	ALA 50	ALA 100		Control	DIC	SLY	ALA 50	ALA 100
ALT (U/L)	36.14^##^ ± 3.53	82.29** ± 6.70	65.71**^##^ ± 6.87	72.14**^##^ ± 3.29	40.71^##^ ± 3.50	Albumin (g/dL)	4.24^##^ ± 0.17	3.24** ± 0.25	3.70**^##^ ± 0.14	3.63**^##^ ± 0.11	3.82**^##^ ± 0.37
AST (U/L)	53.71^##^ ± 3.59	101.43** ± 8.60	72.29**^##^ ± 5.38	72.29**^##^ ± 5.56	58.00^##^ ± 3.06	Globulin (g/dL)	2.98^##^ ± 0.37	1.84** ± 0.45	2.71**^##^ ± 0.44	2.71^##^ ± 0.43	3.19^##^ ± 0.54
ALP (U/L)	134.29^##^ ± 10.40	194.43** ± 9.81	165.29**^##^ ± 9.48	159.43**^##^ ± 8.72	142.29**^##^ ± 10.26	Urea (mg/dL)	29.14^##^ ± 3.72	95.14** ± 5.05	87.29**^##^ ± 4.92	64.14**^##^ ± 6.77	39.57**^##^ ± 4.31
Total bilirubin (μmol/L)	6.10^##^ ± 0.66	9.83** ± 1.75	7.80**^##^ ± 0.52	7.62**^##^ ± 0.53	6.35^##^ ± 0.65	Creatinine (mg/dL)	0.65^##^ ± 0.58	1.64** ± 0.17	1.29**^##^ ± 0.10	1.02**^##^ ± 0.13±	0.76^##^ ± 0.10
Total proteins (g/dL)	7.23^##^ ± 0.33	5.08** ± 0.38	6.42**^##^ ± 0.41	6.34**^##^ ± 0.39	7.00^##^ ± 0.46	Uric acid (mg/dL)	4.49^##^ ± 0.62	19.94** ± 1.07	12.60**^##^ ± 1.14	7.67^##^ ± 1.09±	6.09^##^ ± 0.85

1 Data are expressed as mean ± standard deviation n = 7/group.

* and ** significant difference *vs* control group at P-value ≤ 0.05 and P-value ≤ 0.005, respectively;

# and ## significant difference *vs* DIC group at P-value ≤ 0.05 and P-value ≤ 0.005, respectively; SLY – silymarin at 100 mg/kg body weight (b.w.); ALA 50 – α-lipoic acid at 50 mg/kg b.w.; ALA 100 – α-lipoic acid at 100 mg/kg b.w.

### Oxidative stress biomarkers

The activity of MDA and CAT was significantly elevated, but GSH was depleted in the DIC group’s hepatic and renal tissues, in contrast to the negative control group. Interestingly, the pretreatment with SLY at 100 mg/kg b.w., ALA at 50 mg/kg b.w. and ALA at 100 mg/kg b.w. revealed a significant inhibition of MDA and CAT activity in addition to GSH elevation, which contrasted with the DIC-challenged and hepatorenally unprotected group. Furthermore, the group that received the higher dose of ALA showed the best improvement when compared with the other pretreated groups.

### Histopathological studies

**Fig. 4.**
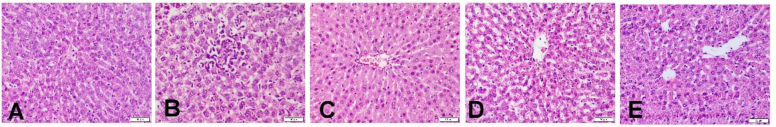
Photomicrographs of haematoxylin and eosin–stained sections of the liver in different experimental groups of Wistar rats subjected to hepatorenal toxic insult by diclofenac sodium (DIC) after being administered or denied protective compounds. A – the liver of a negative control group rat showing normal histological architecture of liver tissue. B – the liver of a DIC-treated and hepatorenally unprotected group rat showing diffused hydropic degeneration of hepatocytes, apoptosis, hepatocellular necrosis and mononuclear infiltration. C – the liver of a silymarin-treated group rat dosed at 100 mg/kg body weight (b.w.) showing hepatocellular regeneration and mild vacuolar degeneration. D – the liver of an α-lipoic acid (ALA)-treated group rat dosed at 50 mg/kg b.w. showing moderate vacuolar degeneration. E – the liver of an ALA-treated group rat dosed at 100 mg/kg b.w. showing marked hepatocellular regeneration, mild cellular necrosis and fatty changes

Haematoxylin and eosin staining and light microscopy were utilised to demonstrate structural histopathological changes in the liver and kidney tissues in various groups. The evaluation of liver tissues from the negative control group showed normal histological architecture (Fig. 4A). The DIC-challenged and hepatorenally unprotected rats exhibited significant histopathological changes, including areas of hydropic degeneration (ballooning) and fatty change (steatosis), along with hepatocellular cytoplasmic microvacuolation, apoptosis, hepatocellular necrosis and mononuclear cell infiltration in central areas (Fig. 4B). Silymarin-treated rats showed signs of hepatocellular regeneration, mild vacuolar degeneration and sporadic hepatocellular necrosis (Fig. 4C). Histopathological investigation of the hepatic tissues from the ALA 50 and ALA 100 groups showed fewer histopathological hepatic alterations (Fig. 4D and E). In the ALA 50 rats, the hepatocytes exhibited moderate vacuolar degeneration, distension from fat vacuoles and congestion of the central vein. The liver samples of rats pretreated with ALA at 100 mg/kg b.w. were free of the previously described hepatic lesions found in DIC-treated and unprotected rats, and cellular regeneration was seen along with a milder degree of cellular necrosis and fatty changes. The scores for histopathological hepatic lesions, including necro-inflammatory changes, are summarised in Fig. 6A, with all treated groups compared with the negative control group. Additionally, the SLY, ALA 50 and ALA 100 groups were compared with the DIC-challenged and hepatorenally unprotected group.

**Fig. 5.**
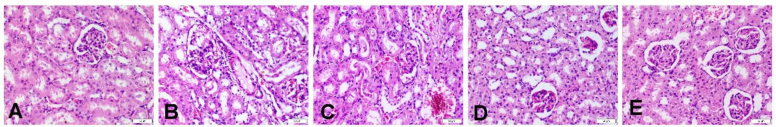
Photomicrographs of haematoxylin and eosin–stained sections of the kidney in different experimental groups of Wistar rats subjected to hepatorenal toxic insult by diclofenac sodium (DIC) after being administered or denied protective compounds. A – the kidney of a negative control group rat showing normal renal architecture with a normal glomerulus and renal tubule arrangement. B – the kidney of a DIC-treated and hepatorenally unprotected group rat showing vacuolisation in the endothelial lining of the glomerular tuft, congestion of renal vessels, desquamation of tubular cells with intraluminal eosinophilic cast formation, severe fatty change in the epithelial lining of renal tubules and necrosis of renal tubules. C – the kidney of a silymarin-treated group rat dosed at 100 mg/kg body weight (b.w.) showing dilation and congestion of renal vessels, tubular cell desquamation and intraluminal cast formation. D and E – the kidneys of α-lipoic acid–treated group rats dosed at 50 mg/kg b.w. or 100 mg/kg b.w. showing marked improvement in renal alterations, restored normal histological structure of the endothelial cell lining of the glomerular tuft and reduced renal necrosis

**Fig. 6.**
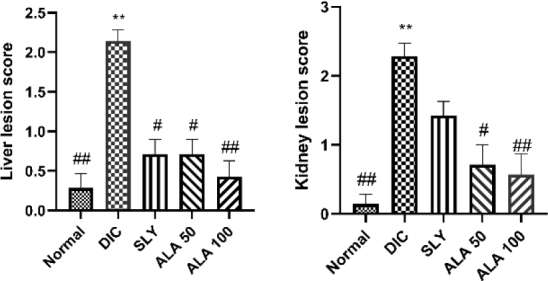
Histopathological evaluation of hepatic and renal lesion scoring for Wistar rat organs after hepatorenal toxic insult with diclofenac sodium (DIC) preceded by administration of protective compounds and contrast in the organs of rats denied the compounds. A – necro-inflammatory score in liver tissue. B – glomerular, interstitial and vascular score in kidney tissue. Data are expressed as median and interquartile range. Adjusted P values were considered significant at P-value ≤ 0.05. DIC – scores for rats administered DIC and not protected hepatorenally; SLY – scores for rats administered silymarin at 100 mg/kg body weight (b.w.) as hepatorenal protection, ALA 50 – scores for rats administered α-lipoic acid at 50 mg/kg b.w. as hepatorenal protection; ALA 100 – scores for rats administered α-lipoic acid at 100 mg/kg b.w. as hepatorenal protection; * and ** – significant difference *vs* normal control group at P-value ≤ 0.05 and P-value ≤ 0.005, respectively; # and ## – significance difference *vs* DIC group at P-value ≤ 0.05 and P-value ≤ 0.005, respectively

The kidney histopathological investigations of the negative control group revealed normal architecture with normal glomerulus and tubular arrangement of the renal tissue (Fig. 5A). The DIC-challenged and hepatorenally unprotected rats revealed dilatation and renal vessels congestion, renal glomerular distortion with vacuolation in the endothelial lining of the glomerular tuft, increased mononuclear cell infiltration of the glomerulus and severe and extensive renal tubular necrosis, with subsequent loss of cellular constituents and desquamation of tubular cells as well as formation of intraluminal eosinophilic cast and severe fatty change in the epithelial lining of renal tubules (Fig. 5B). Silymarin-treated rats showed renal vascular dilation and congestion as well as cellular desquamation and intraluminal cast formation in the tubular cells (Fig. 5C). Histological examinations of the kidneys from ALA 50 and ALA 100 pretreated rats showed none of the histopathological renal alteration; the histological structure of the endothelial cell lining of the glomerular tuft was normal and renal necrosis was less (Fig. 5D and E). The histopathological renal lesion scores, including for glomerular, interstitial and vascular changes, are summarised in Fig. 6B. All the pretreated groups were compared with the negative control group. Additionally, the SLY, ALA 50 and ALA 100 groups were compared with the DIC-challenged and hepatorenally unprotected group.

### Immunohistochemistry of caspase-3 in the liver

**Fig. 7.**
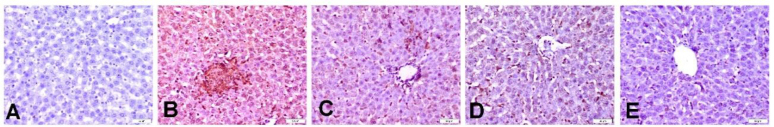
Photomicrographs of caspase-3 activity in livers in different experimental groups of Wistar rats subjected to hepatorenal toxic insult by diclofenac sodium (DIC) after being administered or denied protective compounds. A – the liver of a normal control group rat showing no detectable caspase-3 immunostaining. B – the liver of a DIC-treated and hepatorenally unprotected group rat showing strong caspase-3 immunostaining of hepatocytes undergoing apoptosis. C – the liver of a silymarin-treated group rat dosed at 100 mg/kg body weight (b.w.) showing moderately stained hepatocytes. D – the liver of an α-lipoic acid–treated group rat dosed at 50 mg/kg b.w. showing moderate caspase-3 immunostaining of hepatocytes. E – the liver of an α-lipoic acid–treated group rat dosed at 100 mg/kg b.w. showing a reduction of the immunopositive cells

Immunohistochemical staining was employed to evaluate the expression of liver caspase-3 protein in all the experimental groups. Caspase-3 immunostaining was not detected in the liver tissue in the negative control group (Fig. 7A). The slides of the DIC-challenged and hepatorenally unprotected group revealed strong positive immune expression of caspase-3 protein in the hepatocytes undergoing apoptosis, in contrast to the negative control group (Fig. 7B). The SLY group revealed moderately caspase-3-stained hepatocytes (Fig. 7C). The ALA 50 group showed moderate caspase-3 immunostaining of hepatocytes, and the ALA 100 group showed a reduction of immunopositive cells (Fig. 7D and E). Image analysis of the DIC-treated and unprotected group showed a significant upregulation in caspase-3 immune expression compared with the negative control group (Fig. 9A), which was significantly reversed in the groups pre-treated with either dose of ALA or with SLY. A significant dose-dependent decline in the caspase-3 staining percentage was indicated, comparing ALA 50 with ALA 100 (P-value = 0.0037). Notably, the ALA at dose of 100 mg/kg dose was not statistically different compared with the control, unlike the lower 50 mg/kg dose (Fig. 9A).

### Immunohistochemistry of caspase-3 in the kidney

**Fig. 8.**
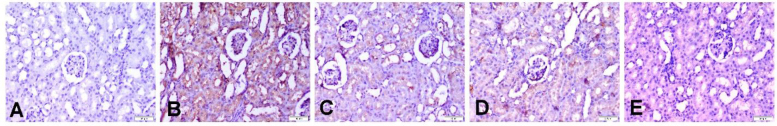
Photomicrographs of caspase-3 activity in kidney in the different experimental groups of Wistar rats subjected to hepatorenal toxic insult by diclofenac sodium (DIC) after being administered or denied protective compounds. A – the kidney of a normal control group rat showing no expression of caspase-3 in the renal corpuscles and tubules. B – the kidney of a DIC-treated and hepatorenally unprotected group rat showing strong positive caspase-3 expression in the glomeruli and tubules. C – the kidney of a silymarin-treated group rat dosed at 100 mg/kg body weight (b.w.) showing moderately stained glomeruli and tubules. D and E – the kidneys of α-lipoic acid–treated group rats dosed at 50 mg/kg b.w. or 100 mg/kg b.w. showing a reduction of the immunopositive cells

**Fig. 9.**
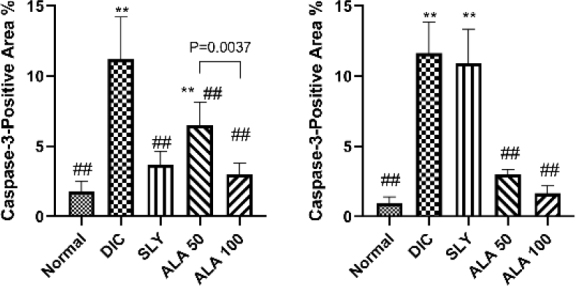
Image analysis data to evaluate the area corresponding to caspase-3 protein expression in the organs of different experimental groups of Wistar rats subjected to hepatorenal toxic insult by diclofenac sodium (DIC) after being administered or denied protective compounds. A – caspase-3 immunostaining expressed as area % in liver tissues. B – caspase-3 immunostaining expressed as area % in kidney tissues. Data are expressed as mean +/− standard deviation. DIC – diclofenac; SLY – silymarin at 100 mg/kg body weight (b.w.); ALA 50 – α-lipoic acid at 50 mg/kg b.w.; ALA-100 – α-lipoic acid at 100 mg/kg b.w.; * and ** – significant difference *vs* normal control group at P-value ≤ 0.05 and P-value ≤ 0.005, respectively; ## – significant difference *vs* DIC group at P-value ≤ 0.005

The immunohistochemical staining revealed that no detectable expression of caspase-3 protein was recorded in the renal corpuscles or tubules of the negative control group (Fig. 8A). Immunohistochemical staining of the DIC-treated and hepatorenally unprotected group kidneys’ slides showed intense caspase-3 staining in the glomeruli, proximal and distal renal tubules, which contrasted with the absence of this in the negative control group (Fig. 8B). Pretreatment with SLY showed that caspase-3 staining of glomeruli and tubules (Fig. 8C) was not statistically significantly more intense than that in the DIC-treated group (Fig. 9B). The results for the ALA 50 and ALA 100 pretreated groups indicated significantly reduced caspase-3 activity, and low caspase-3 expression was recorded in the glomeruli and tubules of both groups (Figs 8D and E, and 9B). Image analysis to evaluate the area corresponding to caspase-3 expression in the DIC-challenged and hepatorenally unprotected group found this expression to be significantly higher in comparison with the negative control group (Fig. 9B). Caspase-3 expression was significantly less in the groups given ALA pretreatment, with no significant difference between the 50 and 100 mg/kg b.w. doses of ALA.

### Effects of ALA on Nrf2/HO-1 and NQO-1 signalling pathway-related genes

**Fig. 10.**
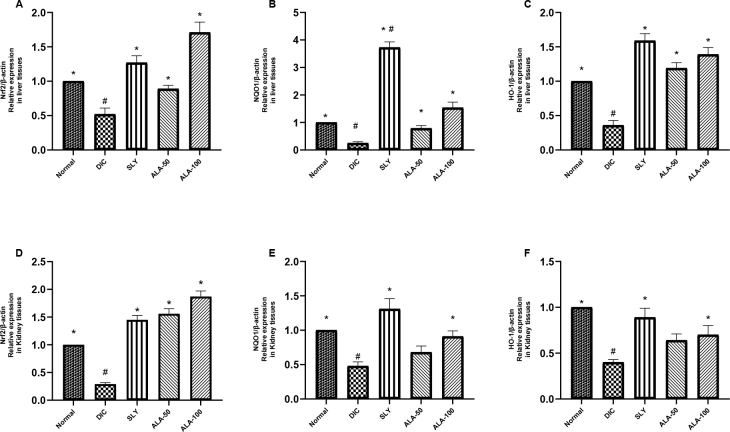
Alpha-lipoic acid (ALA) effects on the messenger RNA expression levels of genes in the liver and kidney tissues of Wistar rats subjected to hepatorenal toxic insult by diclofenac sodium (DIC) after being administered or denied protective compounds. A and D – effect on nuclear factor erythroid 2-related factor 2 (*Nrf2*). B and E – effect on nicotinamide adenine (phosphate) reduced form : quinone oxidoreductase 1 (*NQO1*). C and F – effect on haem oxygenase 1 (*HO-1*). Data are expressed as mean ± standard deviation. n = 7 rats/group; SLY – silymarin at 100 mg/kg body weight (b.w.); ALA 50 – α-lipoic acid at 50 mg/kg b.w.; ALA 100 – α-lipoic acid at 100 mg/kg b.w.; * and ** significant difference *vs* normal control group at P-value ≤ 0.05 and P-value ≤ 0.005, respectively; # – significant difference *vs* DIC group at P-value ≤ 0.05

To further evaluate the mechanism of the protective effects of ALA against DIC-induced hepatorenal toxicity, the primary mRNA species involved in the Nrf2 signalling pathway were assessed by qRT-PCR. At transcriptional levels, DIC-intoxicated and unprotected rats showed a significant downregulation in *Nrf2, NQO-1* and *HO-1* compared with the normal control group. By contrast, supplementation with ALA 50 and ALA 100 upregulated the transcriptional levels of *Nrf2, NQO-1* and *HO-1* in both liver (Fig. 10A–C) and kidney (Fig. 10D–F), doing so statistically significantly more intensely in most cases compared with these genes’ transcriptional levels in the DIC group.

## Discussion

Non-steroidal anti-inflammatory drugs are commonly used for alleviating pain and inflammation. Normal therapeutic doses cause minor side effects, but an overdose of these medications is extremely toxic (23). In the current study, DIC treatment altered the hepatorenal redox status (GSH and CAT activity) and lipid peroxidation (MDA); in addition, it significantly increased serum ALT, AST and ALP activities and total bilirubin, creatinine, urea and uric acid levels. Also, serum total proteins, albumin and globulins were significantly reduced in the DIC-challenged and hepatorenally unprotected group compared with the negative control group. These biochemical markers were aligned with hepatorenal damage noted in the histopathological investigations of the liver and kidney in the current study. Previous studies observed similar deterioration in liver and kidney histopathology associated with DIC toxic insult (6, 11, 23). The liver and kidney dysfunction caused by DIC as demonstrated in the current study is due to toxic compounds excreted during DIC metabolism (28). This hazardous effect can be provoked *via* alterations in mitochondrial permeability, activation of cytochrome P450 and overproduction of ROS (29).

Previous studies confirmed that DIC treatment generated ROS, which exacerbated oxidative status by decreasing antioxidant system activity (6). In the present study, the recorded significant elevation of the hepatorenal lipid peroxidation marker MDA and CAT enzymatic activity as well as the depletion of GSH levels following DIC exposure confirmed the altered hepatorenal redox status. Consistently with the findings of this study, Rian *et al*. (29) recorded significant deterioration of the hepatic oxidative defence mechanism upon DIC exposure. Also, Alabi and Akomolafe (6) reported similar results concerning hepatorenal redox status imbalance after DIC intoxication. Non-steroidal anti-inflammatory drugs have been identified as impacting mitochondria with a disrupting effect on their function. This dysfunction involves impaired oxidative phosphorylation, which leads to excessive ROS production. These, in turn, cause lipid peroxidation, leading to a rise in MDA. Such mechanisms might account for the increase observed within the hepatic and renal tissues of rats receiving an overdose of DIC (6).

Reactive oxygen species overproduction and protective antioxidant molecule depletion (*e.g*. depletion of GSH) are regarded as the most sensitive indicators of drug-induced hepatotoxicity (14). Previous research has shown that increasing DIC dosage could result in GSH depletion, which impairs antioxidant status and promotes toxic metabolites to build up in liver tissue (29). Similarly, DIC has been demonstrated to deplete GSH, exacerbating renal oxidative status (4). The CAT enzyme is one of the most vital antioxidant enzymes and splits two hydrogen peroxide molecules into a single molecule of oxygen and two molecules of water in a two-step reaction (3). In our study, we observed CAT activation in both hepatic and renal tissues. This activation aligns with previous findings suggesting increased CAT activity occurs as a direct consequence of hydrogen peroxide overproduction during DIC metabolism (5, 29).

Cell damage induced by oxidative stress can be caused by modifications to the signalling pathways that regulate gene expression, among which pathways are those for apoptosis, and these modifications raise the likelihood of apoptosis (19). The relative gene expression of the *Nrf2* gene was reduced in the hepatorenal tissues of the DIC group in the present study, demonstrating an alteration in the redox system in this group. The *Nrf2* gene is the master regulator of a set of genes involved in detoxification and antioxidant defences, as well as cryoprotection (19). Furthermore, it has been suggested that Nrf2 may increase cellular susceptibility to oxidative damage by increasing the *HO-1* stress-response gene expression. Consistently with the findings of the current study, Karimi-Matloub *et al*. (23) detected a significant downregulation in renal *Nrf2* and *HO-1* gene expressions due to DIC exposure. Also, Elbaz *et al*. (11) showed a significant reduction in hepatic expression of *Nrf2* together with GSH depletion. Confirmatory histopathological investigations reported higher lesion scoring and stronger expression of caspase-3 in the hepatorenal tissue of the DIC-challenged group. Many cytotoxic compounds cause mitochondrial-dependent apoptosis *via* activation of the caspase family of cysteine proteases. Reactive oxygen species alter the permeability of the mitochondrial membrane, leading to the escape of intermembrane proteins that can initiate several apoptotic pathways (17). Therefore, we speculated that DIC induces hepatorenal damage through the initiation and activation of mitochondrial-dependent apoptotic pathways due to the overproduction of ROS.

In the current study, *NQO1* gene expression showed significant downregulation in both hepatic and renal tissues in DIC groups in comparison with its expression in the control group. This gene has drawn attention because of its crucial cytoprotective role and its activation during cellular stress. Through a two-electron process, NQO1 can directly reduce co-enzyme Q10 (CoQ10) in lipid environments to ubiquinol (30). This, and ubiquinone, the fully oxidised state of CoQ10, other derivatives of CoQ10, Vitamin E quinone and enzymatic reductases are essential to the plasma membrane redox status, and NQO1 plays a significant role in producing reduced forms of CoQ10, preventing lipid peroxidative damage (30). Therefore, we speculated that the downregulation of NQO1 increased the susceptibility of the hepatorenal microenvironment to oxidative damage by DIC insult.

Antioxidant protective mechanisms scavenge excess ROS, by which they mitigate cellular damage induced by increased ROS production. Therefore, antioxidants maintain the balance between the cell signalling oxidant generation, which is beneficial, and the hazardous excess ROS production that induces cellular damage (13).

Pretreatment with ALA either at the low dose (50 mg/kg) or the high dose (100mg/kg) showed the antioxidant properties of the acid by inhibiting DIC causation of oxidative stress in a dose-dependent manner. Alpha lipoic acid, which is crucial for mitochondrial dehydrogenase mechanisms, has recently received a lot of interest as an antioxidant. It can scavenge several ROS, including toxic oxygen species, hypochlorous acid, and peroxyl hydroxyl and superoxide radicals (2). Additionally, by interacting with glutathione and vitamin C, it can afford protection to cell membranes and could potentially recycle vitamin E (26). This hepatorenal protective potential was manifested in a previous study which reported that ALA protected against valproic acid–induced liver damage with its antioxidant and anti-inflammatory properties (26). Alpha lipoic acid has also been proved to prevent renal function impairment, expansion of the glomerular mesangial matrix and glomerulosclerosis by replenishing glutathione and lowering MDA accumulation (10). This organosulphur compound can also efficiently modulate antioxidative enzyme levels and reduce inflammatory response to hepatitis and oxidative deterioration (16). Moreover, it has been reported that ALA consumption significantly decreased MDA levels (12). This could explain the protective effect of ALA against GSH depletion and MDA elevation and thereby the CAT activity reduction in the hepatic and renal tissues in the groups pretreated with ALA before DIC insult. The hepatorenal protection given by ALA is reflected in the amelioration of liver function parameters (AST, ALP, total bilirubin, total proteins, albumin and globulins) and kidney function markers (urea, uric acid and creatinine).

Alpha lipoic acid enhanced the Nrf2 signalling pathway, which potentiated the transcription of the enzymes responsible for GSH production and thus GSH levels (16). In parallel to that, the current study showed significant upregulation of *Nrf2* mediated by ALA treatment in a dose-dependent manner. Pretreatment with ALA at 50 and 100 mg/kg b.w. protected the hepatorenal tissue architecture, as was seen in the histopathological investigations of tissue. Similar results were recorded previously after deltamethrin hepatorenal intoxication of rats (24). Caspase-3 is a strong indicator of cell apoptosis. When a cell is apoptotically activated by a stimulus, it undergoes a series of signalling events that result in variable, permanent morphological alterations, and ultimately dies (18). The apoptosisreducing effect of ALA evaluated in the current study was confirmed in the caspase-3 immunohistochemical staining of hepatorenal tissues, which revealed lower caspase-3 expression in the groups receiving ALA than in the DIC-challenged and unprotected group, confirming the hepatorenal antiapoptotic effect of ALA. The organosulphur compound was also reported to be able to reduce the expression of caspase-3 in methotrexate-damaged lung tissue in rats (8). Similarly, ALA induced liver regeneration by inhibiting apoptosis and reducing caspase-3 expression in liver tissue damaged by a polyunsaturated-fatty-acid-rich diet (24).

The current study revealed significant upregulation of the *Nrf2* gene expression in the ALA-pretreated groups in a dose-dependent manner when compared with DIC-challenged group. Alpha lipoic acid promotes the Nrf2/HO-1 pathway by reducing the interaction of Nrf2 and Keap1. Also, in promoting this pathway, ALA reduces the levels of the inflammatory mediators ROS and IL-8 (1). The protective efficacy of ALA has been ascribed to its impact on redox state and/or energy metabolism. Alpha lipoic acid protects mitochondrial function and improves glucose uptake. Moreover, ALA induces cellular antioxidants, chelates and stabilises metals and directly scavenges reactive oxygen species, which impact the extrinsic pathway of apoptosis and prevent hepatorenal apoptotic progression (10).

## Conclusion

The current study demonstrated that ALA exhibits beneficial protective effects against DIC-induced hepatorenal toxicity. This acid significantly ameliorated the biochemical, histopathological and molecular changes caused by DIC overdose. Specifically, ALA administration resulted in a marked reduction in the oxidative stress biomarkers MDA and ROS, restored the levels of the antioxidants GSH and CAT and improved liver and kidney function, as evidenced by decreased serum ALT, AST, ALP, urea, creatinine and uric acid levels. Histopathological analyses also confirmed the structural preservation of liver and kidney tissues in ALA-pretreated rats. The protective effects of ALA were further supported by the upregulation of the Nrf2 signalling pathway, which plays a significant role in cellular antioxidant defence mechanisms.
